# Mechanisms of the antihypertensive effects of *Nigella sativa* oil in L-NAME-induced hypertensive rats

**DOI:** 10.6061/clinics/2015(11)07

**Published:** 2015-11

**Authors:** Kamsiah Jaarin, Wai Dic Foong, Min Hui Yeoh, Zaman Yusoff Nik Kamarul, Haji Mohd Saad Qodriyah, Abdullah Azman, Japar Sidik Fadhlullah Zuhair, Abdul Hamid Juliana, Yusof Kamisah

**Affiliations:** Universiti Kebangsaan Malaysia, Faculty of Medicine, UKMMC, Department of Pharmacology, Cheras/Kuala Lumpur, Malaysia

**Keywords:** *Nigella sativa*, Antihypertensive, Angiotensin-converting Enzyme, Heme Oxygenase, NADPH Oxidase

## Abstract

**OBJECTIVES:**

This study was conducted to determine whether the blood pressure-lowering effect of *Nigella sativa* might be mediated by its effects on nitric oxide, angiotensin-converting enzyme, heme oxygenase and oxidative stress markers.

**METHODS:**

Twenty-four adult male Sprague-Dawley rats were divided equally into 4 groups. One group served as the control (group 1), whereas the other three groups (groups 2-4) were administered L-NAME (25 mg/kg, intraperitoneally). Groups 3 and 4 were given oral nicardipine daily at a dose of 3 mg/kg and *Nigella sativa* oil at a dose of 2.5 mg/kg for 8 weeks, respectively, concomitantly with L-NAME administration.

**RESULTS:**

*Nigella sativa* oil prevented the increase in systolic blood pressure in the L-NAME-treated rats. The blood pressure reduction was associated with a reduction in cardiac lipid peroxidation product, NADPH oxidase, angiotensin-converting enzyme activity and plasma nitric oxide, as well as with an increase in heme oxygenase-1 activity in the heart. The effects of *Nigella sativa* on blood pressure, lipid peroxidation product, nicotinamide adenine dinucleotide phosphate oxidase and angiotensin-converting enzyme were similar to those of nicardipine. In contrast, L-NAME had opposite effects on lipid peroxidation, angiotensin-converting enzyme and NO.

**CONCLUSION:**

The antihypertensive effect of *Nigella sativa* oil appears to be mediated by a reduction in cardiac oxidative stress and angiotensin-converting enzyme activity, an increase in cardiac heme oxygenase-1 activity and a prevention of plasma nitric oxide loss. Thus, *Nigella sativa* oil might be beneficial for controlling hypertension.

## INTRODUCTION

Hypertension is defined as a persistent elevation of systolic blood pressure of 140 mmHg or greater and/or diastolic blood pressure of 90 mmHg or greater. Hypertension is a major risk factor for cardiovascular, cerebrovascular and renal diseases 1.

Oxidative stress is important in the pathogenesis of essential hypertension or in arterial damage related to essential hypertension 2-3. This was shown by an elevation of lipid peroxidation in hypertensive patients 2. The increases in oxidative stress will reduce the bioavailability of nitric oxide (NO), a potent vasodilator 3 and this reduction contributes to the development of hypertension.

Many factors are involved in blood pressure control, including angiotensin-converting enzyme (ACE), heme oxygenase (HO-1) and the endothelial-derived relaxing factor (EDRF) NO. ACE promotes the production of angiotensin II (Ang II) from angiotensin I (Ang I), which has a direct vasoconstrictor effect on the vessels that increase blood pressure. Ang II has been reported to increase vascular superoxide anion production via membrane NADPH oxidase activation, which will then lead to an increase in lipid peroxidation, inflammation and vascular injury 4-5. HO-1 protects against cardiovascular diseases such as atherosclerosis and hypertension 6. The antihypertensive effect of HO-1 is likely to be mediated by carbon monoxide (CO). CO reduces peripheral resistance by directly dilating blood vessels via the activation of guanylate cyclase 7. CO exerts antihypertensive effects by stimulating the release of NO, reducing sympathetic outflow and promoting sodium excretion via the kidneys 6. Overexpression of the HO-1 gene reduces the Ang II-mediated pressure response in rats 6. However, HO-1 regulates blood pressure by reducing the activity of Ang II, which helps to reduce vascular inflammation and protects against vascular injuries 6. Cardiac-specific expression of HO-1 was shown to protect against inflammation and oxidative damage in healthy subjects 7. Treatment with heme-arginate, an HO-1 inducer, protects the heart by reducing subendocardial injury, interstitial fibrosis and cardiomyocyte hypertrophy, which is believed to occur via a reduction in several inflammatory and oxidative mediators 8.

The use of medicinal plants for the prevention of cardiovascular diseases has been increasing recently. *Nigella sativa*, which is also known as black cumin, has been shown to possess blood pressure-lowering effects in both animals and humans 9-13. It has been used for centuries for medicinal and culinary purposes throughout the Middle East, India and Northern Africa 11. The seed oil of *N. sativa* was found to be rich in thymoquinone and polyphenols 13.

However, the exact mechanism of the blood pressure-lowering effects of *N. sativa* remains uncertain. The antihypertensive effects of *N. sativa* may involve several mechanisms. El Tahir et al. 11 suggested that *N. sativa* likely reduces blood pressure via a reduction in myocardial contractility and heart rate and that *N. sativa* might activate muscarinic receptors on blood vessels. However, Magyar et al. 14 suggested that *N. sativa* might have calcium channel-blocking properties. To the best of our knowledge, the effects of *N. sativa* on ACE, HO-1, NADPH oxidase and NO have not been thoroughly explored. Therefore, the present study was designed to determine the effects of *N. sativa* oil on ACE, HO-1 and cardiac redox status (NADPH oxidase and malondialdehyde) in N_ω_-nitro-L-arginine methyl ester (L-NAME)-treated rats. Nicardipine was used in the study as a positive control because it has antioxidant effects and calcium channel-blocking properties.

## MATERIALS AND METHODS

### Experimental study design

After a week of adaptation, twenty-four male Sprague-Dawley rats (180-220 g) obtained from the Laboratory Animal Resource Unit, Faculty of Medicine, Universiti Kebangsaan Malaysia were divided into four experimental groups. One group served as a control whereas the other three groups were administered L-NAME intraperitoneally (25 mg/kg) 15. Groups 3 and 4 were given both oral *N. sativa* oil (2.5 mg/kg) 16 and oral nicardipine (3 mg/kg), respectively, 17 daily for 8 weeks concomitantly with L-NAME administration. After 8 weeks of treatment, the rats were sacrificed, and their hearts were harvested. Systolic blood pressure was measured weekly by a tail-cuff method (Powerlab, ADInstrument, NSW, Australia). Blood was obtained at weeks 0 and 8 for the determination of plasma nitric oxide. The rats were kept in polyethylene cages in a well-ventilated room at 27° C with a 12-hour light-dark cycle and had free access to water and food throughout the study. All animal management and handling procedures, in addition to the study design, were approved by the university ethical and research committee (Approval No.: 421-January-2012-December-2013).

### Measurement of blood pressure

A non-invasive method was used to measure systolic blood pressure as described by Jaarin et al. 18 using PowerLab data acquisition systems (ADInstruments, Castle Hill, NSW, Australia). A monitoring cuff was placed on the proximal tail to detect changes in blood flow that occurred during occlusion or the release of the cuff. The rats were placed in an approximately body-sized plastic container prior to the blood pressure measurement. This step ensured acclimation and a faster blood pressure measurement. The animals were pre-warmed for 15 minutes to increase blood flow to the tails. A minimum of 5 measurements were recorded and the mean was used for analysis.

### Heart malondialdehyde content measurement

Lipid peroxidation determined as malondialdehyde (MDA) was measured in the cardiac samples following a method by Ledwozyw et al. 19 with some modifications. The protein content in the heart was estimated using an established method 20.

### Heart NADPH oxidase assay

NADPH oxidase activity measured as superoxide production in the cardiac samples was based on a reduction of ferricytochrome c in ferrocytochrome at pH 7.8 as described by Mustapha et al. 21. The samples were homogenized in 0.25 M sucrose (pH 7.8). The reaction mixture containing 250 µg/l cytochrome c, 100 µM NADPH and 50 µg of protein samples was incubated at 37° C for 120 min, either in the presence or absence of diphenyleneiodonium (DPI, 100 µM). The absorbance of the reduction of cytochrome c was read at 550 nm. Superoxide production was calculated from the difference between the absorbance of the samples incubated with and without DPI using an extinction coefficient of 21 mM^−1^cm^−1^.

### ACE and HO-1 assays

ACE (USCN Life, West Lake, Wuhan, China) and HO-1 (Enzo Life Sciences, MI, USA) activities were analyzed in cardiac samples using commercially available kits following the manufacturer's instructions. The colored end products of these enzymes were measured by a microplate reader (Molecular Devices, Sunnyvale, CA, USA) at 450 nm.

### Nitric oxide

The production of NO was indirectly detected by measuring the nitrite (NO_2_-) metabolite content in the samples. Plasma and cardiac NO content were estimated based on a previously described method 22 using Griess reagent (Sigma-Aldrich, St. Louis, MO, USA). Briefly, 50 µg of tissue samples were incubated with the same volume of Griess reagent in a microplate for 5 min in the dark at room temperature. The colored end product was measured using a microplate reader at 540 nm. Plasma NO was expressed as the percentage difference between weeks 0 and 8.

### Statistical analysis

The data are presented as the mean±SEM. The statistical analyses were conducted using Statistical Product for Social Science 16.0 software (SPSS, Inc., Chicago, IL). The normality of the data was evaluated using a Kolmogorov–Smirnov test. Differences between the groups for each parameter were compared using a one-way analysis of variance (ANOVA) with Tukey's Honestly Significant Differences post-hoc test. Statistical significance was defined as *p*<0.05.

## RESULTS

### Effects on systolic blood pressure

**Figure 1:**
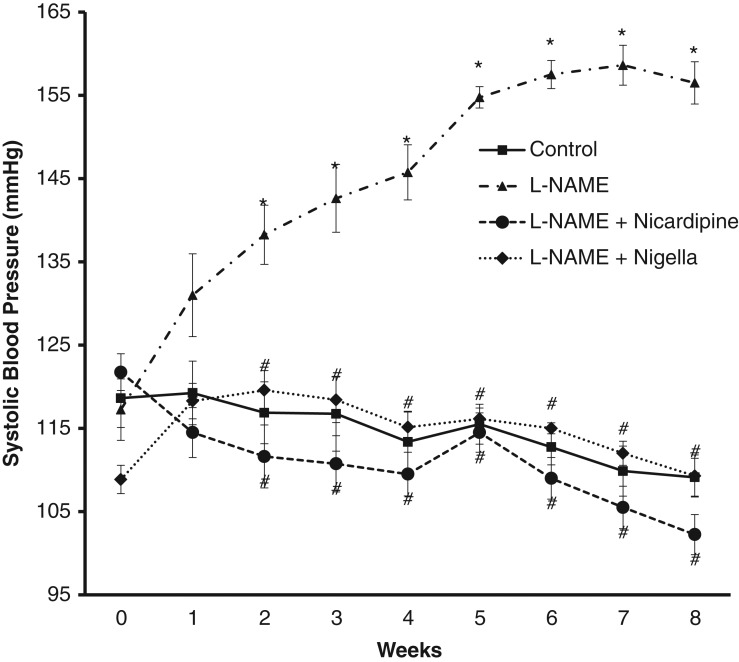
Systolic blood pressure in rats given *N. sativa* oil (NS) and concurrent administration of L-NAME. The data represent the mean±SEM (n=6). *p<0.01 *versus* control, #*p*<0.05 *versus* L-NAME.

There was a significant increase in systolic blood pressure in the L-NAME-treated rats from week 2 to week 8 of the study. Conversely, the treatment of the L-NAME-treated hypertensive rats with *N. sativa* and nicardipine resulted in a significantly lower blood pressure (Figure 1). The blood pressures of the control, *N. sativa-* and nicardipine-treated groups were similar.

### Effect on cardiac MDA

**Figure 2:**
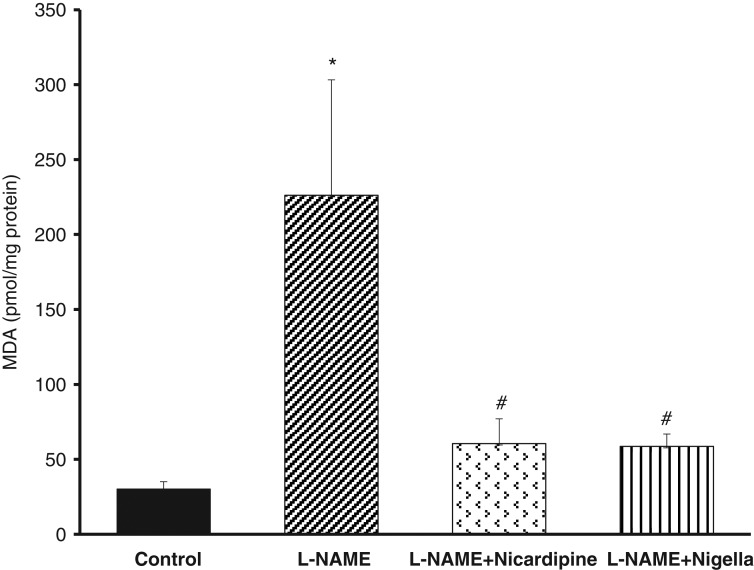
Effect of *N. sativa* oil on heart lipid peroxidation product in rats administered L-NAME for 8 weeks. The bars represent the mean±SEM (n=6). *p<0.01 *versus* control, # *p*<0.05 *versus* L-NAME.

The L-NAME-treated group displayed a significant increase in cardiac MDA content compared to the control. L-NAME+nicardipine and L-NAME+*Nigella sativa* significantly lowered the MDA content compared to the L-NAME alone (*p*<0.05) (Figure 2). There was no significant difference in cardiac MDA content in the control, L-NAME+nicardipine and L-NAME+*Nigella sativa* groups.

### Effect on cardiac NADPH oxidase activity

**Figure 3:**
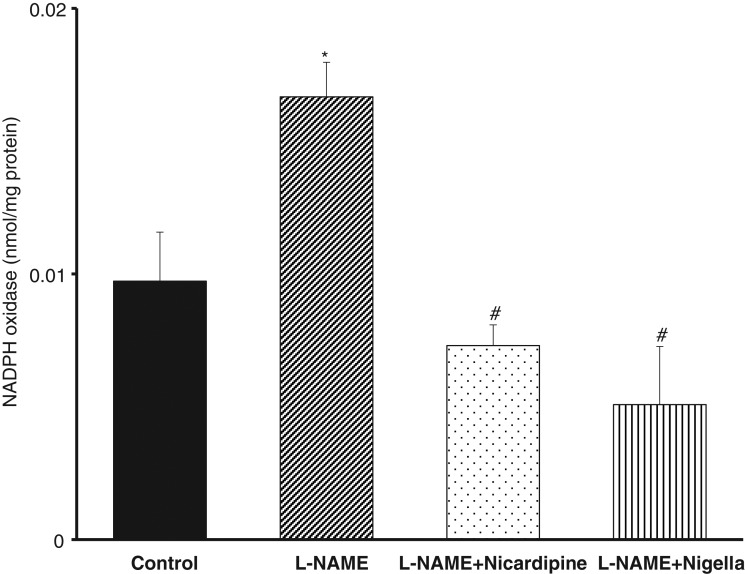
Effects of *N. sativa* oil on NADPH oxidase enzyme activity in L-NAME administered rats. The bars represent the mean±SEM (n=6). *p<0.005 *versus* control, #*p*<0.05 *versus* L-NAME.

A significant elevation of cardiac NADPH oxidase activity was observed in the L-NAME group. The nicardipine- and *N. sativa*-treated groups displayed lower enzyme activity. No significant difference was noted among the control, nicardipine- and *N. sativa*-treated groups (Figure 3).

### Effect on cardiac ACE, HO-1 activities and NO content

L-NAME administration increased cardiac ACE activity compared to control, nicardipine and *N. sativa* treatments. In contrast, enzyme activity was significantly reduced in the nicardipine- and *N. sativa*-treated groups (*p*<0.05) (Table 1). No significant difference was observed in ACE activity between these two groups. The HO-1 activity in cardiac tissues was unaffected by L-NAME. However, the activity of HO-1 was significantly higher in the *N. sativa*-treated group than in the control (*p*<0.005) and nicardipine (*p*<0.05) groups (Table 1). The NO content in the heart was unaffected by either the treatment of L-NAME or the treatment of *N. sativa* (Table 1).

### Effect on plasma NO

**Figure 4:**
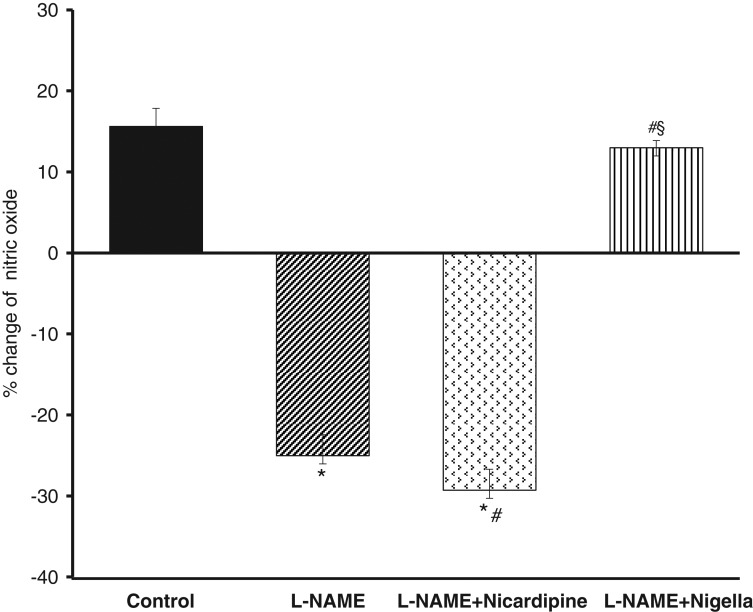
Percentage change of plasma NO (weeks 0 and 8) in rats administered L-NAME and *N. sativa* oil for 8 weeks. The bars represent the mean±SEM (n=6). *p<0.001 versus control, #*p*<0.001 *versus* L-NAME, §*p*<0.001 *versus* nicardipine-treated group.

There was a significant reduction in plasma NO levels in the L-NAME and nicardipine-treated groups compared to the control (*p*<0.001). The NO level was significantly higher in the *N. sativa*-treated group compared to the L-NAME and nicardipine-treated groups (*p*<0.001) (Figure 4).

## DISCUSSION

In this study, we demonstrated that the intraperitoneal administration of an NO synthase inhibitor (L-NAME) at a dose of 25 mg/kg increased blood pressure in rats. This finding was similar to previous studies, which reported that L-NAME at a dose of 40-55 mg/kg per day increased blood pressure 23-24. The blood pressure raising effect of L-NAME in this study was not only associated with a significant decrease in serum NO but also with an increase in stress oxidative biomarkers, including MDA and NADPH oxidase. These findings suggest that the blood pressure-raising effects of L-NAME might not be purely attributed to the inhibition of NO synthase but may involve oxidative stress via the activation of NADPH oxidase expression. Our results were consistent with those of another study, which reported that L-NAME administration at a dose of 0.7 mg/ml in drinking water for 2 weeks enhanced NADPH oxidase expression in aortic tissue 25. Toba et al. 25 reported that L-NAME administration increased oxidative stress, vascular inflammation and ACE activity and expression. Our result suggests that L-NAME may increase blood pressure via the activation of the renin-angiotensin system. This finding was in line with Zanchi et al. 23, who reported that L-NAME might increase blood pressure via activation of the renin-angiotensin system. In this study, L-NAME reduced NO levels in the plasma but not in the cardiac tissues. This finding was contrasted with Bernatova et al. 24, who reported that L-NAME reduced the activity of NO synthase in the left ventricle. Moreover, Ndisang et al. 8 reported that L-NAME-treated rats had increased cyclic guanosine and pro-inflammatory chemokines and cytokines in the heart. The reason for this finding was not clear. We postulated that the negative effect of L-NAME on cardiac NO levels may be a result of the lower dose of L-NAME used in this study, which was 25 mg/kg/day, whereas Bernatova et al. 24 used a higher dose of L-NAME (40 mg/kg/day).

The administration of *N. sativa* oil a dose of 2.5 mg/kg for 8 weeks attenuated the systolic blood pressure increase in the rats that were co-treated with L-NAME. The effect of the oil on blood pressure in this study was in agreement with other studies 9-13. The reduction in systolic blood pressure with *N. sativa* in this study was accompanied by significant reductions in MDA, ACE and NADPH oxidase activities and by an increase in HO-1 activity in cardiac tissue, in addition to an increase in plasma NO. Khattab and Nagi 12 also reported that thymoquinone at a dose of 0.5 mg/kg/day and 1 mg/kg/day orally prevented L-NAME-induced hypertension and renal damage, most likely via an antioxidant effect.

Reductions in L-NAME-induced MDA content and NADPH oxidase activity by the *N. sativa* oil in this study indicate that the oil has an antioxidant effect and may contribute to blood pressure reduction. The antioxidant effects of *N. sativa* have been reported previously 11-13. This finding concurs with Sayed et al. 13 and the Khatab and Nagi 12 studies, which suggested that the antioxidant effect of *N. sativa* contributed towards its antihypertensive effect. The seed oil that contains thymoquinone, dithymoquinone and thymol might contribute to this effect 10,13. Thymol acts as a singlet oxygen quencher whereas thymoquinone and dithymoquinone have superoxide dismutase (SOD)-like activity and also act as free radical scavengers 27-29. Oxidative stress causes endothelial dysfunction, leading to a reduction in the release or production of NO 30-32, the proliferation of vascular smooth muscle cells and collagen deposition, which causes a thickening of the tunica media, and a narrowing of the vascular lumen 28, which in turn impairs vasodilation 30-32. Apart from its antioxidant properties, the blood pressure-lowering effect of *N. sativa* may be attributed to its diuretic properties 33, its anti-inflammatory effect 34 or its reno-protective effect 35.

The activation of HO-1 plays an important role in reducing blood pressure by reducing Ang II-induced inflammation and NADPH oxidase-mediated oxidative stress via the production of anion superoxide 4-6. The antihypertensive effect of HO-1 is attributable to its production of CO, which also has a vasodilator effect 6. In our study, cardiac HO-1 activity was significantly increased in rats that were treated with L-NAME and *N. sativa.* The effect of thymoquinone on the overexpression of HO-1 expression was also reported by Kundu et al. 36.

ACE converts Ang I to Ang II. The reduction of ACE activity leads to a reduction in blood pressure because of the reduction in Ang II synthesis. Ang II acts as a potent vasoconstrictor. Ang II exacerbates oxidative stress by increasing the production of superoxide 4-5. Increased ACE activity and oxidative stress was observed in hypertensive animals 23-25. Increased plasma Ang II availability was reported in L-NAME-treated rats because of endothelial NO synthase (eNOS) inhibition 25. In the current study, *N. sativa* oil reduced cardiac ACE activity in rats receiving L-NAME. This result suggests that the antioxidant properties of *N. sativa* oil contributed to the reduction of the enzyme activity. We are unsure of how the reduction in cardiac ACE reduced blood pressure because our study did not measure serum ACE and aldosterone levels. Further study of serum ACE and aldosterone must be performed in the future. However, a previous study by Zeggwagh et al. 37 demonstrated that *N. sativa* aqueous extract had no effect on plasma ACE activity in spontaneously hypertensive rats.

EDRF, better known as NO, causes vasodilation, which subsequently reduces total peripheral resistance and hence reduces blood pressure. The inhibition of eNOS reduces NO production, whereas eNOS stimulation increases NO production. Our study showed that treatment with *N. sativa* prevented the loss of plasma NO caused by L-NAME. The mechanism on how *N. sativa* reversed the reduction in NO levels in spite of L-NAME was unclear. We postulated that the effect is most likely attributable to the high antioxidant content of *N. sativa*, which helps prevent oxidative stress, vascular inflammation and dysfunction induced by L-NAME. A similar antioxidant effect on NO was reported by Nurul-Iman et al. 38.

The effect of *N. sativa* in this study was comparable to that of nicardipine, which is a short-acting calcium antagonist. We chose nicardipine for this study because it has antioxidant effects and *N. sativa* was reported to possess calcium channel-blocking properties 14. The nicardipine-induced reduction of blood pressure in this study was associated with a reduction in MDA and NADPH oxidase, which indicated that nicardipine has antioxidant properties. The antioxidant effect of a calcium antagonist has been previously reported 39. In this study, similar to *N. sativa*, nicardipine reduced cardiac ACE. The effect of nicardipine on ACE in this study was in accordance with Kataoka et al. 40, who reported that a calcium channel blocker had an effect on the renin-angiotensin system and hypothesized that the anti-inflammatory and antioxidant effects of calcium channel blockers might be a result of the inhibition of the local renin-angiotensin system. The effect of a calcium antagonist on ACE was further supported by Toba et al. 25; they reported that amlodipine prevented the increase in ACE in L-NAME treated rats. However, in this study, nicardipine was not able to inhibit the loss of NO induced by L-NAME treatment. The effect of nicardipine in this study differed from the results of a few other studies that demonstrated that the long-acting calcium antagonists amlodipine and felodipine increased endothelial NO synthase (eNOS) activity and gene expression, respectively and hence increased NO 25. The reason for the discrepancy between our results and those of the other two studies was unclear. We postulated that short- and long-acting calcium channel blockers might have different effects on NO. In this study, we used nicardipine, which belongs to a short-acting dihydropyridine group of calcium antagonists, whereas the other study 25 used a long-acting drug. Further study will be necessary to clarify this discrepancy. Unlike *N. sativa*, nicardipine had no effect on HO-1. In conclusion, *N. sativa* oil at a dose of 2.5 mg/kg attenuates the L-NAME-induced increase in blood pressure and was associated with a reduction in cardiac redox status and angiotensin-converting enzyme activity and an increase in HO-1 activity. *N. sativa* oil also prevented plasma NO loss. Notably, the blood pressure-lowering effect was comparable to that of nicardipine.

## Figures and Tables

**Table 1:** Cardiac angiotensin-converting enzyme, heme oxygenase activities and nitric oxide level.

	ACE (pg/g)	HO (ng/g)	Nitric oxide (µmol/g)
Control	520.27 ± 42.59	8.66 ± 0.45	0.129 ± 0.004
L-NAME	637.40 ± 14.95*	11.69 ± 0.94	0.106 ± 0.011
L-NAME+Nicardipine	483.07 ± 31.29#	9.79 ± 0.84	0.132 ± 0.010
L-NAME+*Nigella*	464.07 ± 31.29#	15.48 ± 2.02*§	0.098 ± 0.008

**<?ENTCHAR ast?>:** *versus* control (*p*<0.05), #*versus* L-NAME (p<0.05)

§*versus* L-NAME+Nicardipine (*p*<0.05).

## References

[1] WHO (2013). A global brief on hypertension: Silent killer, global public health crisis.

[2] Russo C, Olivieri O, Girelli D, Guarini P, Carletto A, Corrocher R (1998). Anti-oxidant status and lipid peroxidation in patients with essential hypertension. J Hypertens.

[3] Kobayashi A, Ishikawa K, Mastsumoto H, Kimura S, Kamiyama Y, Maruyama Y (2007). Synergetic antioxidant and vasodilatory action of carbon monoxide in angiotensin II- induced cardiac hypertrophy. Hypertension.

[4] Chen J, Chen W, Zhu M, Zhu Y, Yin H, Tan Z (2011). Propofol attenuates angiotensin II-induced apoptosis in human coronary artery endothelial cells. Br J Anaesth.

[5] Hong D, Bai YP, Shi RZ, Tan GS, Hu CP, Zhang GG (2014). Inhibitory effect of reinioside C on vascular smooth muscle cells proliferation induced by angiotensin II via inhibiting NADPH oxidase-ROS-ENK1/2-NF-kappaB-AP-1 pathway. Pharmazie.

[6] Durante W (2010). Targeting heme oxygenase-1 in vascular disease. Curr Drug Targets.

[7] Furchgottt RF, Jothianandan D (1991). Endothelium-dependent and independent vasodilatation involving cyclic GMP: relaxation induced by nitric oxide, carbon monoxide and light. Blood Vessels.

[8] Ndisang JF, Chibbar R, Lane N (2014). Heme oxygenase suppresses markers of heart failure and ameliorates cardiomyopathy in L-NAME-induced hypertension. Eur J Pharmacol.

[9] Aftab A, Asif H, Mujeeb M, Khan SA, Najmi AK, Siddique NA (2013). A review on therapeutic potential of <italic>Nigella sativa</italic>: A miracle herb. Asian Pac J Trop Biomed.

[10] Fallah Huseini H, Amini M, Mohtashami R, Ghamarchehre ME, Sadeqhi Z, Kianbakht S (2013). Blood pressure lowering effect of <italic>Nigella sativa</italic> L. seed oil in healthy volunteers: a randomized, double-blind, placebo-controlled clinical trial. Phytother Res.

[11] El Tahir KEH, Al Ajmiand MF, Al-Bekari AM (2003). Some cardiovascular effects of dethymoquinonated <italic>Nigella sativa</italic> volatile oil and its major components α-pinene and p-cymene in rats. Saudi Pharm J.

[12] Khattab MM, Nagi MN (2007). Thymoquinone supplementation attenuates hypertension and renal damage in nitric oxide deficient hypertensive rats. Phytother Res.

[13] Sayed HM, El-Latif HAA, Eid NI, Elsayed AZ, El-Kader EMA (2009). Potential antihypertensive and antioxidative effects of <italic>Nigella sativa</italic> seeds or biomass and <italic>Syzygium aromaticum</italic> extracts on L-NAME-induced hypertensive rats. Egypt J Pharm Sci.

[14] Magyar J, Szentandrassy N, Banyasz T, Fulop L, Varro A, Nasasi PP (2004). Effects of terpenoid phenol derivatives on calcium current in canine and human ventricular cardiomyocytes. Eur J Pharmacol.

[15] Saores Campelo MW, Soares Campelo APB, Franca Lopez LG, Santos AAD, Guimaraes SB, Vanconcelos PRLD (2011). Effects of Rut-bpy (Cis-[Ru(bpy)2 (SO3)(NO)] PF6, anovel nitric oxidedonor, in L-NAME-induced hypertension in rats. Acta Cir Bras.

[16] Houcher Z, Boudiaf KH, Benboubetra M, Houcher B (2007). Effects of methanolic extract and commercial oil of <italic>Nigella sativa </italic>L. on blood glucose and antioxidant capacity in alloxan-induced diabetic rats. Pteridines.

[17] Higuchi S, Sasaki H, Seki T (1980). Pharmacokinetic studies on nicardipine hydrochloride, a new vasodilator, after repeated administration to rats, dogs and humans. Xenobiotica.

[18] Jaarin K, Mustafa MR, Leong XF (2011). The effect of heated vegetables oils on blood pressure in rats. Clinics.

[19] Ledwozyw A, Michalak J, Stepien A, Kadziolka A The relationship between plasma triglycerides, cholesterol total lipids and lipids peroxidation products during human atherosclerosis. Clin Chem Acta.

[20] Lowry OH, Rosebrough NJ, Farr AL, Randall RJ (1951). Protein measurement with the Folin phenol reagent. J Biol Chem.

[21] Mustapha NM, Tarr JM, Kohner EM, Chibber R (2010). NADPH oxidase versus mitochondria-derived ROS in glucose-induced apoptosis of pericytes in early diabetic retinopathy. J Ophthalmol.

[22] Miranda KM, Espey MG, Wink DA (1992). A rapid, simple spectrophotometric method for simultaneous detection of nitrate and nitrite. Nitric Oxide. 2001;5(1):62-71. Baylis C, Mitruka B and Deng A. Chronic blockade of nitric oxide synthesis in rats produces systemic hypertension and glomerular damage. J Clin Invest.

[23] Baylis C, Mitruka B, Deng A (1992). Chronic blockade of nitric oxide synthesis in rats produces systemic hypertension and glomerular damage. J Clin Invest.

[24] Beratova I, Pechanova OG, Simko F (1999). Effect of captopril in L-NAME induced hypertension on rat's myocardium, aorta, brain and kidney. Exp Physiol.

[25] Toba H, Nakagawa Y, Miki H, Shimizu T, Yoshimura A, Inoue R (2005). Calcium channel blockage exhibits anti-inflammatory and anti-oxidative effects by augmentation of endothelial nitric oxide synthase and inhibition of angiotensin converting enzymes in the N-nitro-L arginine methyl ester-induced hypertensive rat aorta: Vosoprotective effects beyond the blood pressure lowering effect of amlodipine and manidipine. Hypertens Res.

[26] Zanchi A, Schaad NC, Osterheld M, Grouzmann E, Nussberger J, Brunner HR (1995). Effects of chronic NO synthase inhibition in rats on renin angiotensin system and sympathetic nervous system. Am J Physiol.

[27] Albrecht EWJA, Stegeman CA, Heeringa P, Henning RH, van Goor H (2003). Protective role of endothelial nitric oxide synthase. J Pathol.

[28] Ahmad S, Beg ZH (2014). Mitigating role of thymoquinone rich fractions from <italic>Nigella sativa</italic> oil and its constituents, thymoquinone and limonene on lipidemic-oxidative injury in rats. Springerplus.

[29] Mansour MA, Nagi MN, El-Khatib AS, Al-Bekairi AM (2002). Effects of thymoquinone on antioxidant enzyme activities, lipid peroxidation and DT-diaphorase in different tissues of mice: a possible mechanism of action. Cell Biochem Funct.

[30] Ng CY, Kamisah Y, Faizah O, Jaarin K (2012). The role of repeatedly heated soybean oil in the development of hypertension in rats: association with vascular inflammation. Int J Exp Path.

[31] Ng CY, Kamisah Y, Faizah O, Jaarin K (2012). The role of repeatedly heated soybean oil in the development of hypertension in rats: association with vascular inflammation. Int J Exp Path.

[32] Chan CK, Zhao Y, Liao SY, Zhang YL, Lee MY, Xu A (2013). A-FABP and oxidative stress underlie the impairment of endothelium-dependent relaxations to serotonin and the intima-medial thickening in the porcine coronary artery with regenerated endothelium. ACS Chem Neurosci.

[33] Zaoui A, Cherrah Y, Lacaille-Dubois A, Settaf A, Amarouch H, Hassar M (2000). Diuretic and hypotensive effect of <italic>Nigella sativa</italic> on the spontaneous hypertensive rats. Therapie.

[34] Alagawany M, El-Hack MEA, Faraq MR, Tiwari R, Dhama K (2015). Biological effects and modes of action of carvacrol in animal and poultry production and health. A review. Adv Anim Vet Sci.

[35] Elsherbiny NM, El-Sherbiny M (2014). Thymoquinone attenuates doxorubicin- induced nephrotoxicity in rats. Chem Biol Interact.

[36] Kundu J, Kim DH, Kundu JK, Chun KS (2014). Thymoquinone induces heme oxygenase-1 expression in HaCaT cells via Nrf2/ARE activation: Akt and AMPKα as upstream targets. Food Chem Toxicol.

[37] Zeggwagh NA, Moufid A, Khaldi A, Michel JB, Eddouks M (2009). Cardiovascular effect of <italic>Nigella sativa</italic> aqueous extract in spontaneously hypertensive rats. In: Chemistry and Medicinal Value.

[38] Nurul-Iman, Kamisah Y, Jaarin K, Qoddriyah HMS (2013). Virgin coconut oils prevent elevation of blood pressure and improve endothelial function in rats fed repeatedly heated palm oil. Evid Based Complement Alternat Med.

[39] Allanore Y, Borderie D, Lemaréchal H, Ekindjian OG, Kahan A (2004). Acute and sustained effects of dihydropyridine-type calcium channel antagonists on oxidative stress in systemic sclerosis. Am J Med.

[40] Katoaka C, Egashira K, Ishibashi M (2004). Novel anti-inflammatory action of amlodipine in a rat model of atherosclerosis induced by long term inhibition of nitric oxide synthesis. Am J Physiol Heart Circ Physiol.

